# Phytomedical investigation of Najas minor All. in the view of the chemical constituents 

**DOI:** 10.17179/excli2014-662

**Published:** 2015-03-30

**Authors:** Marina D. Topuzovic, Ivana D. Radojevic, Milan S. Dekic, Niko S. Radulovic, Sava M. Vasic, Ljiljana R. Comic, Braho Z. Licina

**Affiliations:** 1 Department of Biology and Ecology, Faculty of Science, University of Kragujevac, Kragujevac, Serbia; 2 Department of Chemistry, Faculty of Science and Mathematics, University of Niš, Niš, Serbia; 3 Department of Chemical and Technological Sciences, State University of Novi Pazar, Novi Pazar, Serbia; 4 Department of Biomedical Sciences, State University of Novi Pazar, Novi Pazar, Serbia

**Keywords:** Najas minor, antimicrobial, antibiofilm, total phenolic content, flavonoid and tannin content, volatile constituents

## Abstract

Plants are an abundant natural source of effective antibiotic compounds. Phytomedical investigations of certain plants haven't still been conducted. One of them is *Najas minor (N. minor)*, an aquatic plant with confirmed allelopathy. Research conducted in this study showed the influence of water and ethyl acetate extracts of *N. minor* on microorganisms, in the view of chemical profiling of volatile constituents and the concentrations of total phenols, flavonoids and tannins. Antimicrobial activity was defined by determining minimum inhibitory and minimum microbicidal concentrations using microdilution method. Influence on bacterial biofilm formation was performed by tissue culture plate method. The total phenolics, flavonoids and condensed tannins were determined by Folin-Ciocalteu, aluminum chloride and butanol-HCl colorimetric methods. Chemical profiling of volatile constituents was investigated by GC and GC-MS. Water extract didn't have antimicrobial activity below 5000 µg/mL. Ethyl acetate extract has shown strong antimicrobial activity on G+ bacteria - *Staphylococcus aureus *PMFKGB12 and *Bacillus subtilis *(MIC < 78.13 µg/mL). The best antibiofilm activity was obtained on *Escherichia coli *ATCC25922 (BIC50 at 719 µg/mL). Water extract had higher yield. Ethyl acetate extract had a significantly greater amount of total phenolics, flavonoids and tannins. As major constituent hexahydrofarnesyl acetone was identified. The ethyl acetate extract effected only G+ bacteria, but the biofilm formation of G-bacteria was suppressed. There was a connection between those* in vivo* and *in vitro* effects against pathogenic bacterial biofilm formation. All of this points to a so far unexplored potential of *N. minor.*

## Introduction

*Najas minor* All. 1785 Fl. Pedem. 2:221 belongs to the family Najadaceae. *N. minor* is aquatic, submersed, invasive plant that grows in fresh water (calm waters such as rivers, slow-flowing aquatic ecosystems, ponds and lakes) and it's widespread in central and southern Europe, northern and tropical Africa and Asia. It is usually found in depths of 0.6-4.5 m and prefers to live in moderately warm waters (8 °C at the coldest) and can live in the hot summer water too. Stem is to 40 cm long with branched shells. The leaves are opposite, narrow and toothed, up to 2 cm, and the flowers are a unipolar, individually or few in axils list. It's a true annual, grows anew from seeds each spring. Seeds form in the leaf axils from July through September (Јosifović, 1974[7]). 

Relevant literature review has shown little data about the biological activity of *N. minor*. The most commonly study is its allelopathic effect in the community where it is located. The allelopathic effect of different extracts of *N. minor* have been studied on other plants (El-Shahawy, 2012[4]) or some harmful algae (He et al., 2008[5]; Wang et al., 2010[16]). 

Since the other biological activities of *N. minor* have not been investigated so far, the aim of this study was this plant's antimicrobial and antibiofilm activity applying *in vitro *methods, also the determination of total phenol, flavonoid and tannin content from water and ethyl acetate extracts. For seeing the substances possibly responsible for the activity, the chemical profiling of volatile constituents of *Najas minor* ethyl acetate extract has been set. 

## Materials and Methods

### Chemicals 

All chemicals were commercially available and used as received. A Mueller-Hinton broth, which was used as nutrient liquid medium, was obtained from Liofilchem (Italy); Sabouraud dextrose broth from Torlak (Belgrade, Serbia); an antibiotic, tetracycline from Sigma Chemicals Co. (St. Louis, MO, USA) and antimycotic, fluconazole, was purchased from Pfizer Inc. (USA).

### Plant material

In June-July 2012, of whole plant *N. minor* were collected, in Međuvršje Reservoir, central Serbia (position: 43°51´- 43°56´ N, 17°47´- 17°55´ E, altitude: 273m). Identification and classification of the plant material was performed at the Institute of Botany and Botanical garden “Jevremovac”, University of Belgrade (No. 16758). Voucher specimens are deposited in Herbarium of the Institute of Botany and Botanical garden “Jevremovac”, University of Belgrade (BEOU) (Thiers, 2013[13]; continuously updated). The collected plant material was air-dried in darkness at ambient temperature.

### Preparation of plant extracts 

Dried, ground plant material was extracted by maceration with water and ethyl acetate. Firstly, we immersed 50 g of plant material in 250 mL of solvent. Then, the plant material was moistened by fresh solvent at ambient temperature on a daily basis - one time for three days. Afterwards, thus gained filtrates were combined and then put into rotary evaporator at 40 °C to dry. Final extracts were held in sterile sample tubes and conserved at -20 °C. 

### In vitro antimicrobial assay

Table 1(Tab. 1) shows the list of tested microorganisms. Complete clinical isolates were donated by the Institute of Public Health in Kragujevac. Other microorganisms used were from the collection of Microbiology Laboratory of Faculty of Science, University of Kragujevac.

The direct colony method was used in preparation of bacterial and yeast suspensions. The regulation of initial suspension turbidity was conducted by comparison with 0.5 McFarland's standard (Andrews, 2005[2]). Around 10^8 ^colony forming units (CFU)/mL are held within initial bacterial suspensions. In addition, 1:100 dilutions of initial suspension were made into sterile 0.85 % saline. The process of preparing the suspensions of fungal spores included gentle stripping of spore from agar slants with growing aspergilli. The outcome were the suspensions 1:1000 diluted in sterile 0.85 % saline.

The process of testing antimicrobial activity included determination of minimum inhibitory concentration (MIC) and minimum bactericidal concentration (MBC) which was conducted by microdilution method with resazurin (Sarker et al., 2007[12]). Tested plant extracts were dissolved in 10 % DMSO to a concentration of 10000 µg/mL. Sterile 96-well plates were used for making twofold serial dilutions of plant extracts with liquid media for microorganisms. The range of tested concentration was from 5000 to 78.13 µg/mL. Detailed description of this method is presented in previous paper (Vasić et al., 2012[15]). As positive controls we used tetracycline for bacteria and fluconazole for fungi. As negative control we used 10 % DMSO which did not influence the growth of microorganism. Growth control and sterility control were present in all tests. Also, the tests were carried out in duplicate and MICs were constant.

### Tissue culture plate method (TCP)

The TCP assay described by Christensen et al., (1985[3]) is most widely used test for detection of biofilm formation. We screened all strains for their ability to form biofilm by TCP method with some modifications. Each test included biofilm formation control. Bacterial biofilm formation properties were well described by O'Toole et al., (2000[8]). Anti-biofilm activity was calculated in form of the biofilm inhibitory concentration (BIC) at 50 and 90 percent.

The tissue culture 96-well plates (Sarstedt) were filled with Mueller-Hinton broth for bacteria at 50 μL per well. Then extract stock solution (10000 μg/mL), that was tested, was filled in plate's first row and twofold serially diluted. A 50 μL of fresh bacterial suspension was added to each well. Initial bacterial suspensions contain about 10^8^ CFU/mL. 1:100 dilutions of initial suspension were additionally prepared into Mueller-Hinton broth. The obtained extract concentration range was from 5000 to 78 µg/mL. After 24 h at 37 °C of incubation, for inoculated plates, the plates were leisurely tapped in order to remove the present content. Sterile 0.85 % saline was used for washing the wells and removing the unattached bacteria. Empty plates containing only biofilms were filled with 0.1 % w/v crystal violet solution for staining and incubated at the room temperature for 20 minutes. Excess stain was rinsed off thorough washing with deionized water and plates were fixed with 200 µL of ethanol. Optical densities (OD) of stained adherent bacteria were determined with a micro ELISA plate reader at wavelength of 630 nm (OD630 nm). Only broth or broth with extracts served as control to check sterility and non-specific binding of media. All test values were reduced by OD numbers read for sterile medium with extracts (fixed and dyed) in order to recompense the background absorbance.

### Phytochemical analysis 

#### Determination of total phenolic content 

Folin-Ciocalteu's method was used in determining the concentrations of total phenols (Wootton- Beard et al., 2011[17]). The preparation of samples was performed in triplicate and the mean value of absorbance was calculated. Gallic acid was used as a standard for calibration of standard curve. The linear equation of standard curve (y = 0.008x + 0.0077, R2 = 0.998) was used in calculating the total phenolic content. Finally, the total phenolic content was shown by form - milligram of gallic acid equivalent per gram of extract (mg of GAE/g of extract).

#### Determination of total flavonoid content 

Aluminium chloride method was used in determination of the concentrations of flavonoids (Quettier-Deleu et al., 2000[10]). The preparation of samples was performed in triplicate and the mean value of absorbance was calculated. Rutin was used as a standard for calibration of standard curve. The linear equation of standard curve (y = 0.021x + 0.040, R2 = 0.999) was used in calculating the concentrations of total flavonoids. Finally, the concentrations of total flavonoids were shown by form - milligram of rutin equivalent per gram of extract (mg of RU/g of extract).

#### Determination of condensed tannins (proanthocyanidins) 

Butanol-HCl method was used in determination of condensed tannins (Porter et al., 1986[9]). The preparation of samples was performed in triplicate and the mean value of absorbance was calculated. Cyanidin chloride was used as a standard for calibration of standard curve. The linear equation of standard curve (y ¼ 0. 0094 x þ 0.006, R2 ¼ 0.999) was used in calculating the concentrations of proanthocyanidins. Finally, the concentrations of proanthocyanidins were shown by form - milligram of cyanidin chloride equivalent per gram of extract (mg of CChE/g of extract).

### GC-FID and GC-MS analyses

Chemical composition of the extract was conducted by GC and GC-MS. The GC-MS analyses (in triplicate) were carried out on a HP 6890N gas chromatograph coupled with a 5975C mass selective detector using a DB-5 capillary column (30 m × 0.25 mm, film thickness 0.25 μm, Agilent Technologies, USA). The injector and interface temperature were 250 and 320 °C, respectively, while the oven temperature was programmed from 70 to 315 °C at a rate of 5 °C/min and then held isothermally for 10 min. Helium was used as a carrier gas at flow rate of 1.0 ml/min, while 1 μl of the extract solutions were injected in a pulsed split mode in a split ratio of 40:1. The MS was operated at ionization energy of 70 eV in a acquisition mass range of 35-650 amu, with a scanning period of 0.34 s. The same experimental conditions as described above were used for the GC-FID analyses.

The extract constituents were identified

1. by linear retention indices matching (calculated relative to the retention times of C8-C34 n-alkanes (Van den Dool and Kratz, 1963[14]) to those reported in the literature (Adams, 2007[1]),
2. by comparison of mass spectra to those of authentic standards, as well as those from Wiley 6, NIST05 and MassFinder 2.3 library and homemade MS library with the spectra corresponding to pure compounds and
3. by coinjection, wherever possible, with an authentic sample. The percentage composition was obtained by integration of the GC-FID peak area without the use of correction factors.

### Data analysis 

All data were presented as means ± standard deviations (mean ± SD) where appropriate. All statistical analyses were performed using Microsoft Excel software.

## Results

The results of *in vitro* antimicrobial activity of two extracts from *N. minor* against 27 strains of bacteria and fungi, with control results, determined by microdilution method, is presented in Table 1(Tab. 1). 

The intensity of antimicrobial action varied depending on the type of plant extract and on the groups of microorganisms. MICs and MMCs values were in range from < 78.13 µg/mL to > 5000 µg/mL. Water extract didn't had antimicrobial activity below 5000 µg/mL. The ethyl acetate extract has shown the strongest antimicrobial activity on G+ bacteria while the activities on other species were moderate. The tested extract showed high antibacterial activity against species from the genus *Bacillus*. MICs values were in range from < 78.13 µg/mL to 625 µg/mL. Significant effect extract showed in food spoilage isolate* Staphylococcus aureus*, and clinical isolate and standard strain of *S. aureus*. The influence on G- bacteria was either very low or was not observed at all within the tested concentrations (MIC and MMC ranged from 5000 μg/mL to > 5000 μg/mL). The influence of tested extracts on fungi was generally weak. The exception is the ethyl acetate extract on the species *Aspergillus restrictus* (MIC and MMC were < 78.13 µg/mL).

Ethyl acetate extract had influenced the formation of bacterial biofilm (Table 2(Tab. 2)). The maximum concentrations of extract where the biofilm could develop were interesting. 

In this case *Escherichia coli *ATCC 25922 biofilm formation was the most sensitive of all tested strains (BIC90 at 1262 µg/mL) and the less sensitive was *Pseudomonas aeruginosa* ATCC 27853 in forming the biofilm (BIC90 at 6111 µg/mL).

The percent yields of crude extracts, concentration of total phenolic and flavonoid obtained from *N. minor* are presented in Table 3(Tab. 3). Although with noticeably lower yield than water extract, ethyl acetate extract has in itself a significantly greater amount of total phenolics and flavonoids. In Table 3(Tab. 3) is presented concentration of tannins in the both extracts of *N. minor*. As with the previous (total phenols) much higher amount of the tannins is in the ethyl acetate extract.

Table 4(Tab. 4) lists the identified volatile constituents of *N. minor* ethyl acetate extract by means of a detailed GC and GC/MS. The analyses allowed the identification of 23 volatile compounds, accounting for 87.3 % of the detected GC peak areas. The most abundant constituents were hexahydrofarnesyl acetone, (*E*)-phytol and neophytadiene (isomer I) (17.6, 11.9 and 10.1 %, respectively).

Since, until now, antibiofilm and antimicrobial activity was not performed, as well as the content of total phenols, flavonoids and tannins in the extracts of *N. minor,* obtained results can be considered in relation to the different chemical compounds isolated from this plant. It is noticed that the ethyl acetate extract had no effect below 5000 µg/mL in MIC for G-bacteria (Table 1(Tab. 1)). The results have pointed that the ethyl acetate extract had microdilution effect only on G+ bacteria, probably because of the difference in cell wall structure, but the biofilm formation of G-bacteria was suppressed (Table 2(Tab. 2)).

These results are showing the potential of this allelopathically confirmed plant. We have noticed that there is some connection between those* in vivo *effects and *in vitro* effects against biofilm formation by pathogenic bacteria that were tested in this investigation. It can be a starting point for further examination.

Significant antimicrobial activity on most G+ bacteria can be attributed to the action of hexahydrofarnesyl acetone. Hexahydrofarnesyl acetone, the most abundant volatile in our extract, was already proposed to be a potential antimicrobial agent (Radulović et al., 2006[11]). The same compound was also the main volatile constituent of the essential oil isolated from the aquatic plant *Sagittaria trifolia* (Xiangwei et al., 2006[18]). The antimicrobial assays of the essential oil isolated from this plant also showed the significant antimicrobial activity on G+ bacteria (Xiangwei et al., 2006[18]). Some of the terpenoids identified in our sample, such as phytol, have been reported in the literature for their recognized antimicrobial properties (Inoue et al., 2005[6]). The terpenoids also could be responsible and contribute to the observed activity since it was known that these compounds possess the antimicrobial properties.

Other authors by studying allelopathic activity of *N. minor* also found that the water extract had significantly less activity than methanol extract (El-Shahawy, 2012[4]) or ethyl acetate fraction (Wang et al., 2010[16]).

Water and methanol extracts isolated from the whole plant of *N. minor* showed significant allelopathic effect in suppressing seedling root and shoot growth of the assayed species (*Lolium perenne, Corchorus olitorius* and *Amaranthus viridis*) (El-Shahawy, 2012[4]). The results of the other study showed that the ethyl acetate fraction of *N. minor* aqueous extracts had activity to inhibit the growth of *Microcystis aeruginosa* (Wang et al., 2010[16]).

## Conclusion

Although the antimicrobial and antibiofilm activity of *N. minor* have not been investigated so far, the study showed that this plant has notable antimicrobial effect on G+ bacteria and that it reduces the forming of biofilm at G- bacteria. Ethyl acetate extract had great amounts of phenols and basic volatile substance was identified as hexahydrofarnesyl acetone. All of this points to a so far unexplored potential of *N. minor.*

## Acknowledgements

This investigation was supported by the Ministry of Education, Science and Technological Development of the Republic of Serbia, grant No. III 41010, OI 173032 and OI172061. The authors declare that they have no conflict of interest.

## Figures and Tables

**Table 1:**
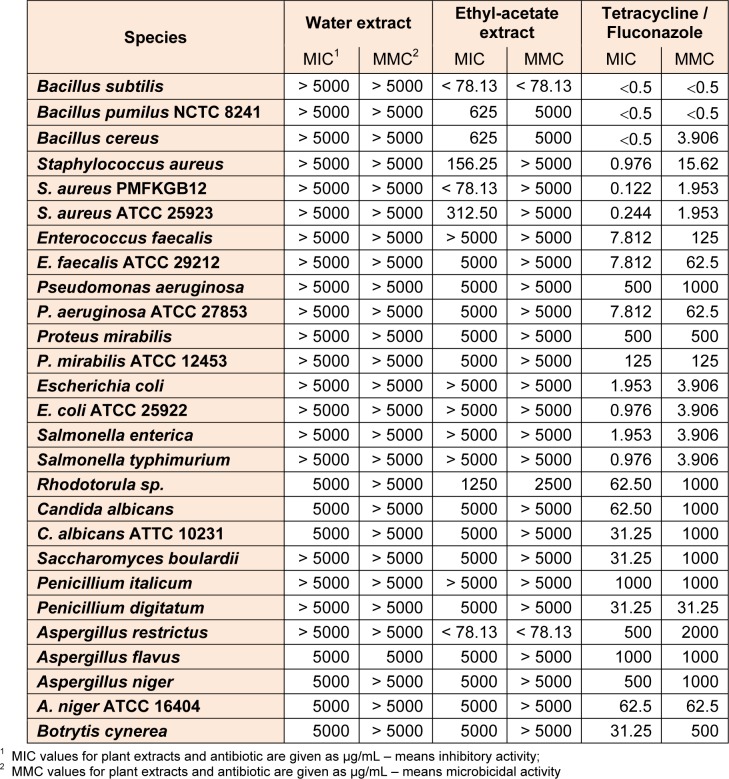
Antimicrobial activity of water and ethyl acetate extracts of *N. minor* against tested microorganisms based on microdilution method

**Table 2:**
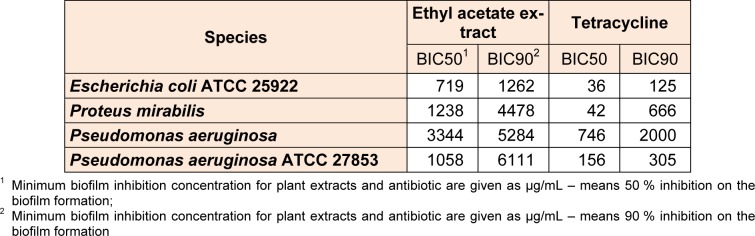
Antibiofilm activity of ethyl acetate extracts of *N. minor* against tested microorganisms

**Table 3:**
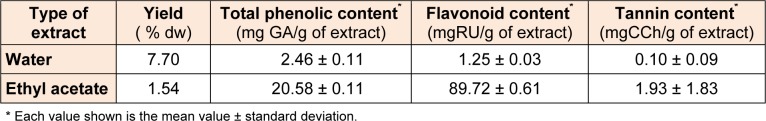
Yield, concentration of total phenolic, flavonoid and tannin content in the extracts of *N. minor*

**Table 4:**
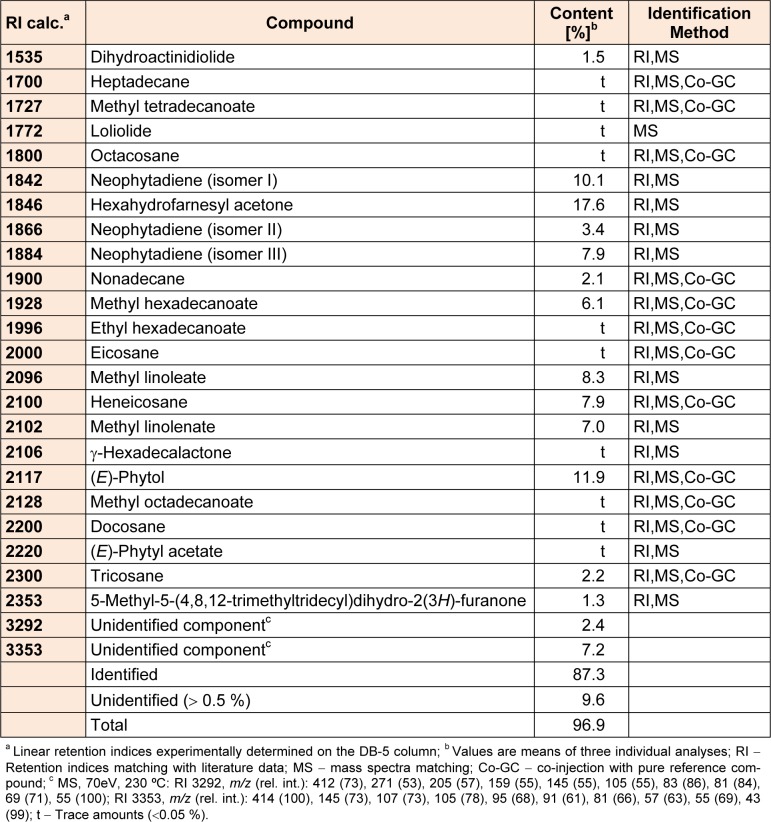
Volatile constituents of *N. minor* ethyl acetate extract
